# Supplementary material for: Artemisinin-independent inhibitory activity of Artemisia sp. infusions against different Plasmodium stages including relapse-causing hypnozoites
Source: Life Sci Alliance. 2021 Dec 2;5(3):e202101237. doi: 10.26508/lsa.202101237 (PMC8675911; doi:10.26508/lsa.202101237)
Supplement: Supplementary file 1 [file LSA-2021-01237_TableS1.docx]

**Supplementary materials**

**Table S1. Quantification of artemisinin in *A. annua* and *A. afra* infusion by UHPLC-MS.**

| **Stock *Artemisia* Infusion** | **Artemisinin concentration in stock infusion** | **Artemisinin concentration in the infusion used for hepatic cultures** |
| --- | --- | --- |
| 100 g *A. annua* leaves and twigs/L | 6.07 ± 0.01 µg/mL  = 21.5 ± 0.03 µM | 0.607 µg/mL (from a 10 g/L infusion)  = 2.15 µM |
| 50 g *A. afra* leaves and twigs/L | *ca.* 0.071 ± 0.007 µg/mL = 251 ± 25 nM  (< limit of quantification) | *ca.* 0.0028 µg/mL (from a 4 g/L infusion*) = 10 nM  (< limit of quantification) |

*****4 g/L infusion was prepared from a 100 g/L stock *A. afra* infusion.
